# Development and Validation of Real-Time PCR for Detecting *Anaplasma bovis*–Like Agent in *Dermacentor* spp. Ticks

**DOI:** 10.3201/eid3205.251750

**Published:** 2026-05

**Authors:** Rachel C. Smith, Daniel F. Barrantes Murillo, Aryanna Carr, Kathryn T. Duncan, Lindsay A. Starkey

**Affiliations:** Oklahoma State University College of Veterinary Medicine, Stillwater, Oklahoma, USA

**Keywords:** Anaplasma bovis, bacteria, zoonoses, real-time PCR, vector-borne infections, tickborne infections, Dermacentor variabilis, Dermacentor andersoni, Dermacentor albipictus, United States

## Abstract

We developed and validated a real-time PCR to detect an *Anaplasma bovis*–like agent. We applied that assay to 672 *Dermacentor* spp. ticks collected from across the United States and found 0.1% *A. bovis–*like agent prevalence. This assay could enhance epidemiologic surveys for this *A. bovis–*like agent in ticks and humans.

An *Anaplasma bovis*–like agent was detected in humans in the United States in 2020 and was further described from 4 patients and a few *Dermacentor* spp. ticks in 2023 (*1*,*2*). Clinical data from human cases was not available, although patient samples were submitted for suspicion of tickborne illness (*1*,*2*). A subsequent regional survey detected the *A. bovis*–like agent in 10.1% (4/38) of *D. variabilis* ticks from Oklahoma but not in 93 Kansas *D. variabilis* ticks or 140 Oklahoma beef cattle (*3*). 

Human illness caused by *A. bovis* has been described in China, where infection of ruminants is common (*4*,*5*). Epidemiologic investigation of the *A. bovis*–like agent is obstructed by lack of a specific, rapid molecular assay. Previous studies have relied on conventional PCRs (*2*,*3*), which are suitable for genetic characterization but cumbersome for screening large sample sets. To improve diagnostic methods, we developed and optimized a real-time probe-based PCR assay specifically for the *A. bovis*–like agent.

## The Study

We retrieved from GenBank heat shock chaperon gene (*gro*EL) sequences from the *A. bovis–*like agent and related *Anaplasma* spp. bacteria, including *A. bovis*, *A. capra*, *A. centrale*, *A. marginale*, *A. ovis*, *A. phagocytophilum*, and *A. platys* (Appendix Table). We aligned sequences and generated potential primers and probes in Geneious Prime version 2025.2.2 (https://www.geneious.com) by using the primer design tool. We generated 1 assay (AboviTM) targeting a 125-bp fragment of the *gro*EL gene. AboviTM satisfied the design parameters, and we expected it to be specific to *A. bovis–*like agent because of base mismatches with nontarget sequences within the oligo binding sites. 

We had custom AboviTM oligos synthesized in TaqMan assays (Thermo Fisher Scientific, https://www.thermofisher.com), then further interrogated the oligos (Table 1). We performed PCR reactions in MicroAmp EnduraPlate Optical 96-Well Clear Reaction Plates on an Applied Biosystems QuantStudio 3 Real-Time PCR instrument (both Thermo Fisher Scientific) and included nontemplate controls for every run.

**Table 1:** *O*ligo sequences used to develop and validate real-time PCR for detecting *Anaplasma bovis*–like agent in *Dermacentor* spp. ticks, United States*

Type	Oligo name	Sequence, 5′ → 3′
Primer	AboviTMF	TGC GCA GTG TGT TAA GGA AG
Primer	AboviTMR	ACG GAG AAA GAT ATC CAC GAT CA
Probe	AboviTMPro	FAM-TCG GAA GAG ACG GAG TAA-MGB

*Oligos devised from heat shock chaperon gene (groEL) sequences from A. bovis–like agent and related Anaplasma spp. bacteria retrieved from GenBank (Appendix Table).

We initially verified AboviTM by amplifying (×3) four *A. bovis–*like DNA samples previously extracted from *D. variabilis* ticks, then confirming by conventional PCR (*3*). Each 20-μL PCR reaction contained 10 μL TaqMan Fast Advanced Master Mix (Thermo Fisher Scientific), 900 nmol of each primer, 250 nmol probe, 2 μL DNA template, and molecular grade water. Thermocycling conditions were enzyme activation at 95°C for 60 seconds, followed by 45× cycles of denaturation at 95°C for 3 seconds, and annealing/extension at 60°C for 30 seconds. To confirm appropriate amplicon size and identity, we performed gel electrophoresis in 2.0% agarose gel, followed by PCR product purification using the Wizard SV Gel and PCR Clean-up Kit (Promega, https://www.promega.com). Oklahoma State University (OSU) Molecular Core Facility performed Sanger sequencing (unidirectional), and we confirmed sequence identity by BLAST analysis (https://blast.ncbi.nlm.nih.gov).

For further assay optimization, we used a gBlocks synthetic double-stranded DNA standard (Integrated DNA Technologies, https://www.idtdna.com) of the target sequence. We prepared a series of 10-fold dilutions to attain a range of 1–100,000,000 copies of target DNA per reaction. We evaluated combinations of varying primer (75–900 nmol) and probe (100–250 nmol) concentrations by using 100 copies/reaction. We considered assay performance to be optimal when the amplification curve demonstrated concurrently high baseline-corrected normalized reporter and low cycle threshold value (*6*). Assay performance was optimal at primer concentrations of 900 nmol each and probe concentration of 250 nmol. We performed subsequent PCR reactions with those concentrations and the thermocycling protocol described above.

We conducted standard curve analyses to evaluate the AboviTM assay efficiency and sensitivity by using 2 sample sets: the 10-fold dilution series containing synthetic target DNA only and the same dilution series with ≈54 ng of noninfected *D. variabilis* tick DNA added per reaction. We used tick samples to assess assay performance in the tick DNA matrix and obtained noninfected adult ticks from the OSU Tick Rearing Facility. 

**Figure 1:**
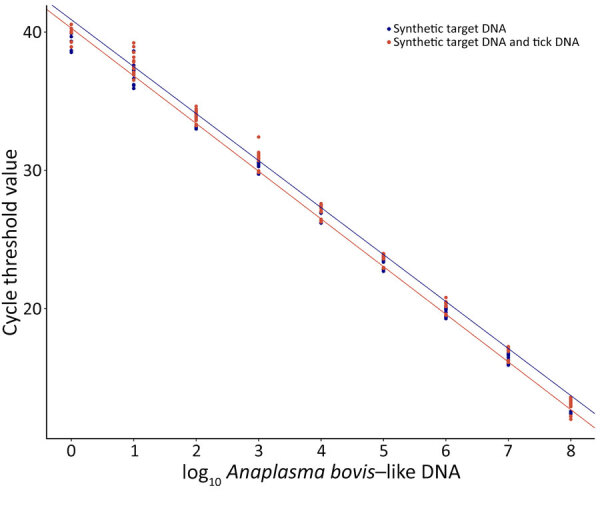
Results of the standard curve analyses, correlation, and amplification efficiency of real-time PCR for detecting *Anaplasma bovis*–like agent in *Dermacentor* spp. ticks, United States. We conducted standard curve analyses to evaluate assay efficiency and sensitivity by using 2 sets of samples: a 10-fold dilution series containing synthetic *A. bovis*–like DNA only and the same dilution series with ≈54 ng of noninfected *D. variabilis* tick DNA added per reaction. We used tick samples to assess assay performance in the tick DNA matrix. Graph shows standard curve lines for each sample set, with cycle threshold value graphed as a function of target copy number.

We amplified both dilution series in triplicate and repeated that experiment over 5 independent runs. We checked raw PCR data for quality, then used Applied Biosystems Design & Analysis Software version 2.8.0 (Thermo Fisher Scientific) to analyze data and exported data into Excel Version 1808 (Microsoft, https://www.microsoft.com). We calculated standard curve regression, amplification efficiency, and correlation by using the qPCRtools package in R Studio 2024.09.1 (*7*). We used the ggplot2 package in R Studio to visualize statistical and cycle threshold data (Figure 1). 

The assay’s limit of detection was 10 target copies. AboviTM detected as low as 1 target copy; however, limit of detection <3 copies is considered invalid because of stochastic limitations of quantitative PCR (*8*). We interrogated specificity by running AboviTM in triplicate with genetically similar agents that might be detected in ticks or mammals and observed no cross-amplification against *Anaplasma phagocytophilum*, *A. marginale*, *A. platys*, *Ehrlichia ewingii*, *E. chaffeensis*, *Rickettsia bellii*, or *R. montanensis*. Those nontarget DNA samples were previously extracted from ticks or mammalian whole blood and confirmed by PCR and Sanger sequencing (data not shown).

**Figure 2:**
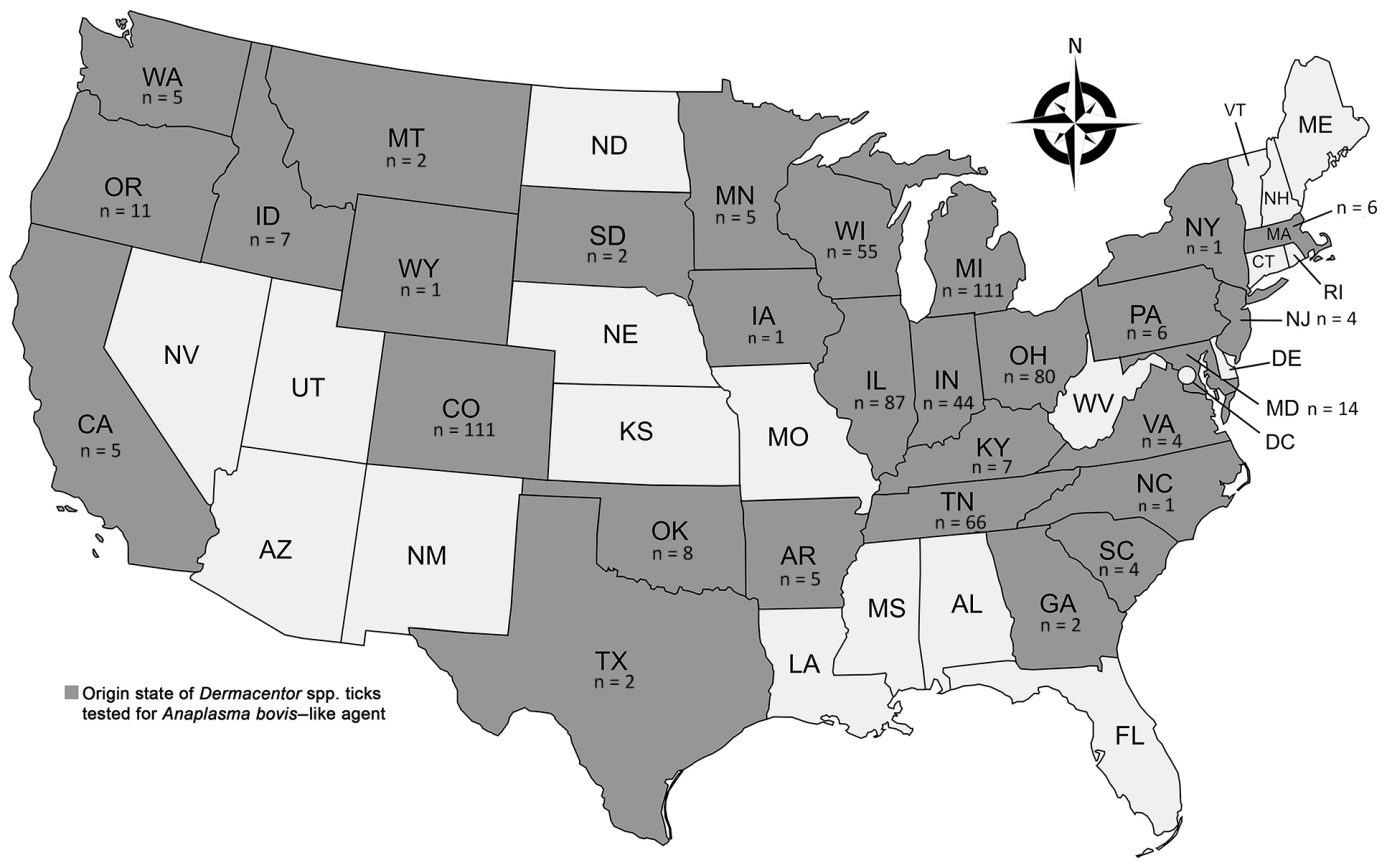
Number and geographic distribution of *Dermacentor* spp. ticks collected from dogs and cats and tested by real-time PCR (AboviTM assay) for *Anaplasma bovis–*like agent, United States. Ticks were collected at veterinary clinics across the country during 2021–2023 and shipped to the Oklahoma State University College of Veterinary Medicine Show Us Your Ticks program (https://www.showusyourticks.org), where staff morphologically identified ticks and stored in 70% ethanol at −20°C until dissection and DNA extraction in this study.

We then applied the optimized AboviTM assay to 672 *Dermacentor* spp. ticks collected from dogs and cats across the United States during 2021–2023 (Figure 2). Veterinary clinics shipped ticks to the OSU College of Veterinary Medicine (https://www.showusyourticks.org), where staff morphologically identified ticks and stored in 70% ethanol at −20°C until dissection and DNA extraction. 

We performed dissection by adhering ticks, ventral side down, on glass slides using double-sided adhesive tape. We used sterile no. 11 scalpels to open each tick along the coronal plane and to scrape out internal contents, which we transferred to sterile 1.5 mL microfuge tubes. We discarded tick exoskeletons. We used Cytiva Blood Genomic Prep Mini Spin Kit (Cytiva, https://www.cytivalifesciences.com) to extract total genomic DNA from tick samples, according to manufacturer recommendations. We tested <1 female and <1 male tick per species from an individual host. We detected *A. bovis–*like agent from only 1 (0.1%) tick (Table 2): a male *D. variabilis* tick collected from a dog in Blaine, Minnesota. We further verified that positive sample by using conventional PCRs targeting the 16S (*rrs*), citrate synthetase (*glt*A), and *gro*EL genes, as previously described (*2*,*9*,*10*). We resolved amplicons by gel electrophoresis, then purified, performed Sanger sequencing (unidirectional), and analyzed as described. All sequences were 100% identical to previously reported sequences from *Dermacentor* spp. ticks and humans in the United States (GenBank accession nos. OQ772255, OQ772257.1, OQ693619, and PQ166304–7).

**Table 2:** Results from survey of ticks tested with real-time PCR for detecting *Anaplasma bovis*–like agent in *Dermacentor* spp. ticks, United States*

Tick species	Tick host			Tick sex		No. ticks tested	No. PCR positive (% [95% CI])
	Dog	Cat		F	M		
*D. variabilis*	592	39		394	237	631	1 (0.2 [0–0.9])
*D. andersoni*	35	2		30	7	37	0 (0 [0–9.5])
*D. albipictus*	2	2		3	1	4	0 (0 [0–60.2])
Total	629	43		427	245	672	1 (0.1 [0–0.8])

*Ticks were collected at veterinary clinics across the United States during 2021–2023 and shipped to the Oklahoma State University College of Veterinary Medicine Show Us Your Ticks program (https://www.showusyourticks.org), where staff morphologically identified ticks and stored in 70% ethanol at −20°C until dissection and DNA extraction in this study.

## Conclusions

Few cases of human *A. bovis–*like infection have been reported in the United States (*1*,*2*). Some data suggest *Dermacentor* spp. tick transmission, although we detected *A. bovis–*like DNA in only 0.1% of *Dermacentor* spp. ticks tested. In our previous survey, we detected *A. bovis–*like DNA in 10.1% (4/38) of *D. variabilis* ticks from Oklahoma, 3 (75%) of which originated from the same collection site (*3*). Distribution of *A. bovis* might be highly focalized because, including findings from this study, *A. bovis–*like DNA has only been detected in humans and *D. variabilis* ticks from central US states, including Minnesota, Oklahoma, Missouri, Iowa, and Nebraska, and in *D. andersoni* ticks from Saskatchewan, Canada (*2*,*3*). Other possible tick and mammal species involved in transmission are currently unknown. 

Because *A. bovis–*like agent has been detected in few *Dermacentor* spp. ticks and humans and few studies have investigated its epidemiology, broad conclusions cannot yet be drawn about its complete geographic distribution. AboviTM enables rapid, specific detection for *A. bovis–*like agent with high efficiency, dramatically improving methods for screening samples. Application of AboviTM to future studies could enable further largescale screening of humans, potential vectors, and animal hosts, which could greatly improve epidemiologic understanding of *A. bovis–*like agent in the United States.

Appendix Additional information on development and validation of real-time PCR for detecting *Anaplasma bovis*–like agent in *Dermacentor* spp. ticks.
