# Supplementary material for: Development and Validation of Real-Time PCR for Detecting Anaplasma bovis–Like Agent in Dermacentor spp. Ticks
Source: Emerg Infect Dis. 2026 May;32(5):745–8. doi: 10.3201/eid3205.251750 (PMC13175086; doi:10.3201/eid3205.251750)
Supplement: Appendix — Additional information on development and validation of real-time PCR for detecting Anaplasma bovis–like agent in Dermacentor spp. ticks. [file 25-1750-Techapp-s1.pdf]

Article DOI: https://doi.org/10.3201/eid3205.251705

EID cannot ensure accessibility for supplementary materials supplied by authors. Readers who have difficulty accessing supplementary content should contact the authors for assistance.

Development and Validation of Real-Time PCR for Detecting *Anaplasma bovis*–Like Agent in *Dermacentor* spp. Ticks

Appendix

Appendix Table. GenBank accession numbers for chaperon gene (*gro*EL) sequences from *Anaplasma bovis*–like agent and related Anaplasmataceae used in this study

Sequence type	Source	GenBank accession nos.
A. bovis–like	*Dermacentor variabilis* (tick)	OQ772255
	Dermacentor andersoni (tick)	OQ772257
	Homo sapiens (mammal)	OQ693619
	Dermacentor variabilis (tick)	PQ166304–7
Other Anaplasmataceae		
A. bovis	*Procyon lotor* (mammal)	JN588562
	Amblyomma triguttatum (tick)	KY425440
	Capra hircus (mammal)	MH255908
Anaplasma capra	*Hydropotes inermis* (mammal)	LC432178
Anaplasma centrale	*Cervus elaphus* (mammal)	HM057223
Anaplasma marginale	*Bos taurus* (mammal)	AF165812
Anaplasma ovis	*Haemaphysalis concinna* (tick)	OM648132
Anaplasma phagocytophilum	*Sylvilagus floridanus* (mammal)	DQ088133
	Ixodes ricinus (tick)	MK341070
Anaplasma platys	*Canis lupus familiaris* (mammal)	AF399916
	Canis lupus familiaris (mammal)	AY044161
